# Safety and efficacy of intravenous thrombolysis in stroke patients on prior antiplatelet therapy in the WAKE-UP trial

**DOI:** 10.1186/s42466-020-00087-9

**Published:** 2020-11-20

**Authors:** Benedikt M. Frey, Florent Boutitie, Bastian Cheng, Tae-Hee Cho, Martin Ebinger, Matthias Endres, Jochen B. Fiebach, Jens Fiehler, Ian Ford, Ivana Galinovic, Alina Königsberg, Josep Puig, Pascal Roy, Anke Wouters, Tim Magnus, Vincent Thijs, Robin Lemmens, Keith W. Muir, Norbert Nighoghossian, Salvador Pedraza, Claus Z. Simonsen, Christian Gerloff, Götz Thomalla, on behalf of the WAKE-UP investigators, Götz Thomalla, Christian Gerloff, Amir Golsari, Anna Alegiani, Bastian Cheng, Christoph Beck, Chi-un Choe, Dieke Voget, Julia Hoppe, Julian Schröder, Martin Ebinger, Karl Georg Häusler, Michal Rozanski, Alexander H. Nave, Christian Wollboldt, Ivana Galinovic, Isabelle van Sloten, Jos Göhler, Juliane Herm, Jan Jungehülsing, Janos Lückl, Jan M. Kröber, Johannes Schurig, Luzie Koehler, Ludwig Schlemm, Michael Knops, Maria Roennefarth, Nils Ipsen, Peter Harmel, Rouven Bathe-Peters, Robert Fleischmann, Ramanan Ganeshan, Rohat Geran, Simon Hellwig, Sein Schmidt, Serdar Tütüncü, Thomas Krause, Verena Gramse, Joachim Röther, Peter Michels, Dominik Michalski, Johann Pelz, Andreas Schulz, Carsten Hobohm, Christopher Weise, Gesa Weise, Johannes Orthgieß, Katrin Pomrehn, Mirko Wegscheider, Anne K. Mueller, Michael Hennerici, Martin Griebe, Angelika Alonso, Alexandra Filipov, Annika Marzina, Bettina Anders, Christian Bähr, Carolin Hoyer, Christopher Schwarzbach, Claudia Weber, Eva Hornberger, Hans-Werner Pledl, Matti Klockziem, Markus Stuermlinger, Matthias Wittayer, Marc Wolf, Nadja Meyer, Philipp Eisele, Susanne Steinert, Tamara Sauer, Valentin Held, Peter Ringleb, Simon Nagel, Roland Veltkamp, Sönke Schwarting, Alexander Schwarz, Christoph Gumbinger, Christian Hametner, Hemasse Amiri, Jan Purrucker, Mareva Ciatipis, Oliver Menn, Sibu Mundiyanapurath, Simon Schieber, Tobias Kessler, Tilman Reiff, Verena Panitz, Oliver Singer, Christian Foerch, Arne Lauer, Anika Männer, Alexander Seiler, Damla Guerzoglu, Jan H. Schäfer, Katharina Filipski, Matthias Lorenz, Natalia Kurka, Pia Zeiner, Waltraud Pfeilschifter, Rainer Dziewas, Jens Minnerup, Christian Albiker, Martin Ritter, Matthias Seidel, Ralf Dittrich, Bernd Kallmünzer, Tobias Bobinger, Dominik Madzar, David Stark, Jochen Sembill, Kosmas Macha, Klemens Winder, Lorenz Breuer, Martin Koehrmann, Maximilian Spruegel, Stefan Gerner, Sebastian Moeller, Peter Kraft, Daniel Mackenrodt, Christoph Kleinschnitz, Ahmed Elhfnawy, Florian Heinen, Ignaz Gunreben, Sven Poli, Ulf Ziemann, Alexandra Gaenslen, Dennis Schlak, Florian Haertig, Francesca Russo, Hardy Richter, Matthias Ebner, Martin Ribitsch, Martin Wolf, Christian Weimar, Vesna Zegarac, Hsin-Chieh Chen, Katharina Althaus, Hermann Neugebauer, Eric Jüttler, Julia Meier, Sebastian Stösser, Volker Puetz, Ulf Bodechtel, Claus Z. Simonsen, Leif Ostergaard, Anette Møller, Dorte Damgaard, Kristina Hougaard Dupont, Marika Poulsen, Niels Hjort, Noelia Ruiz de Morales, Paul von Weitzel, Thomas Harbo, Jacob Marstrand, Andreas Hansen, Hanne Christensen, Karen Aegidius, Lise Jeppesen, Per Meden, Sverre Rosenbaum, Helle Iversen, Christina Back, Jakob Hansen, Lene Michelsen, Thomas Truelsen, Boris Modrau, Bo Kristensen, Karsten Vestergaard, Lorenz Oppel, Robin Lemmens, Vincent Thijs, Anke Wouters, Bart Swinnen, Ide Smets, Jelle Demeestere, Laurens Dobbels, Raf Brouns, Ann De Smedt, Jacques DeKeyser, Laetita Yperzeele, Robbert-Jan Van Hooff, Andre Peeters, Anne Dusart, Ana Etexberria, Bernard Hanseeuw, Frederic London, Jonathan Leempoel, Katharina Hohenbichler, Nadia Younan, Vicky Maqueda, Patrice Laloux, Beatrijs De Coene, Charlotte De Maeseneire, Gaetane Turine, Yves Vandermeeren, Nina De Klippel, Chris Willems, Isabel de Hollander, Peter Soors, Sylvia Hermans, Dimitri Hemelsoet, Philippe Desfontaines, Peter Vanacker, Matthieu Rutgers, Charlotte Druart, Perrine Paindeville, Dirk Peeters, Bart Bruneel, Evelien Vancaester, Frederik Vanhee, Guy Meersman, Paul Bourgeois, Patrick Vanderdonckt, Norbert Nighoghossian, Tae-Hee Cho, Amandine Benoit, Laurent Derex, Laura Mechthouff, Nadia Berhoune, Thomas Ritzenthaler, Pierre Amarenco, Cristina Hobeanu, Elena Meseguer Gancedo, David Calvet, Alexandre Ladoux, Alexandre Machet, Catherine Lamy, Charles Mellerio, Catherine Oppenheim, Christine Rodriguez-Regent, Eric Bodiguel, Guillaume Turc, Julia Birchenall, Laurence Legrand, Ludovic Morin, Myriam Edjali-Goujon, Oliver Naggara, Soulliard Raphaelle, Sylvie Godon-Hardy, Valérie Domigo, Vincent Guiraud, Yves Samson, Anne Leger, Charlotte Rosso, Flore Baronnet-Chauvet, Sophie Crozier, Sandrine Deltour, Marion Yger, Igor Sibon, Pauline Renou, Sharmilla Sagnier, Mathieu Zuber, Ruben Tamazyan, Gilles Rodier, Nathalie Morel, Sandra Felix, Wilfried Vadot, Valerie Wolff, Adriana Aniculaesei, Bertrand Yalo, Daniela Bindila, Veronique Quenardelle, Karine Blanc-Lasserre, Emmanuelle Landrault, Ludovic Breynaert, Serkan Cakmak, Stéphane Peysson, Alain Viguier, Claire Lebely, Nicolas Raposo, Anne-Evelyne Vallet, Pauline Vallet, Sébastian Brugirard, Keith W. Muir, Bharath Cheripelli, Dheeraj Kalladka, Fiona Moreton, Krishna Dani, Salwa El Tawil, Sankaranarayanan Ramachandran, Xuya Huang, Elisabeth Warburton, Nicholas Evans, Richard Perry, Bhavni Patel, Geoffrey Cloud, Anthony Pereira, Barry Moynihan, Caroline Lovelock, Lillian Choy, Usman Khan, Christine Roffe, Pippa Tyrell, Craig Smith, Anand Dixit, Stephen Louw, David Broughton, Ashit Shetty, Jason Appleton, Nikola Sprigg, Salvador Pedraza Gutierrez, Boris Raul Acosta, Cecile van Eendenburg, Josep Puig Alcántara, Joaquin Serena Leal, Maria del Mar Castellanos Rodrigo, Mikel Terceno Izaga, Olga Belchí Guillamon, Juan Arenillas, Ana Calleja, Elisa Cortijo, Patricia Mulero, Natalia Pérez de la Ossa, Alicia Garrido, Alicia Martinez, Carlos García Esperón, Cristina Guerrero, David Carrera, Dolores Vilas, Elena Lopez-cancio, Ernest Palomeras, Giuseppe Lucente, Meritxell Gomis, Irina Isern, Juan L. Becerra, Jose Hervas Vicente, Josep Sánchez, Laura Dorado, Laia Grau, Lourdes Ispierto, Luis Prats, Miriam Almendrote, María Hernández, Marta Jimenez, Manuel Lozano Sánchez, Monica Millán Torne, Silvia Presas, Xavier Ustrell, Anna Pellisé, Irene Navalpotro, Alain Luna, Wouter Schonewille, Paul Nederkoorn, Charles Majoie, Lucie van den Berg, Sophie van den Berg, Thomas Zonneveld, Michel Remmers, Franz Fazekas, Alexander Pichler, Simon Fandler, Thomas Gattringer, Johannes Mutzenbach, Jörg Weber, Elmar Höfner, Heinz Kohlfürst, Karin Weinstich, Lars Kellert, Anna Bayer-Karpinska, Christian Opherk, Frank Wollenweber, Matthias Klein, Tobias Neumann-Haefelin, Alexandra Pierskalla, Andreas Harloff, Jürgen Bardutzky, Florian Buggle, Jörg von Schrader, Rainer Kollmar, Josef Schill, Anna-Mareike Löbbe, Thierry Moulin, Benjamin Bouamra, Louise Bonnet, Emmanuel Touzé, Anne-Laure Bonnet, Emmanuel Touze, Julien Cogez, Lin Li, Sophie Guettier, Arindam Kar, Aravinth Sivagnanaratham, Olivia Geraghty, Urszula Bojaryn, Arumugam Nallasivan, Miguel Blanco Gonzales, Manuel Rodríguez-Yáñez, Jose Tembl, David Gorriz, Stefan Oberndorfer, Elisabeth Prohaska

**Affiliations:** 1 Klinik und Poliklinik für Neurologie, Kopf- und Neurozentrum, University Medical Center Hamburg-Eppendorf, Martinistr. 52, 20246 Hamburg, Germany; 2 Hospices Civils de Lyon, Service de Biostatistique, F-69003 Lyon, France; 3 Université Lyon 1, F-69100 Villeurbanne, France; 4 CNRS, UMR 5558, Laboratoire de Biométrie et Biologie Evolutive, Equipe Biostatistique-Santé, F-69100 Villeurbanne, France; 5 Department of Stroke Medicine, Université Claude Bernard Lyon 1, CREATIS CNRS UMR 5220-INSERM U1206, INSA-Lyon, Hospices Civils de Lyon, Lyon, France; 6 Centrum für Schlaganfallforschung Berlin (CSB, Charité - Universitätsmedizin Berlin, Campus Mitte, Charitéplatz 1, 10117 Berlin, Germany; 7 Neurologie der Rehaklinik Medical Park Humboldtmühle, An der Mühle 2-9, 13507 Berlin, Germany; 8 Klinik und Hochschulambulanz für Neurologie, Charité-Universitätsmedizin Berlin, Campus Mitte, Charitéplatz 1, 10117 Berlin, Germany; 9 Department of Diagnostic and Interventional Neuroradiology, University Medical Center Hamburg-Eppendorf, Martinistr. 52, 20246 Hamburg, Germany; 10 Robertson Centre for Biostatistics, University of Glasgow, University Avenue, Glasgow, G12 8QQ UK; 11 Department of Radiology, Institut de Diagnostic per la Image (IDI), Hospital Dr Josep Trueta, Institut d’Investigació Biomèdica de Girona (IDIBGI), Parc Hospitalari Martí i Julià de Salt - Edifici M2, 17190 Salt, Girona, Spain; 12 Department of Neurology, University Hospitals Leuven, Herestraat 49, 3000 Leuven, Belgium; 13 Department of Neurosciences, Experimental Neurology, KU Leuven – University of Leuven, Oude Markt 13, bus 5005, 3000 Leuven, Belgium; 14 VIB, Center for Brain & Disease Research, Laboratory of Neurobiology, Campus Gasthuisberg, Herestraat 49, bus 602, 3000 Leuven, Belgium; 15 Florey Institute of Neuroscience and Mental Health, University of Melbourne, 245 Burgundy Street, Heidelberg, VIC 3084 Australia; 16 Department of Neurology, Austin Health, 145 Studley Road, Heidelberg, VIC 3084 Australia; 17 Institute of Neuroscience & Psychology, University of Glasgow, University Avenue, Glasgow, G12 8QQ UK; 18 Department of Neurology, Aarhus University Hospital, 8200 Aarhus N, Denmark

**Keywords:** Ischemic stroke, Alteplase, Thrombolysis, Recombinant human tissue plasminogen activator, Rt-PA, Aspirin, Clopidogrel, Hemorrhagic transformation, Antiplatelet, WAKE UP

## Abstract

**Background:**

One quarter to one third of patients eligible for systemic thrombolysis are on antiplatelet therapy at presentation. In this study, we aimed to assess the safety and efficacy of intravenous thrombolysis in stroke patients on prescribed antiplatelet therapy in the WAKE-UP trial.

**Methods:**

WAKE-UP was a multicenter, randomized, double-blind, placebo-controlled clinical trial to study the efficacy and safety of MRI-guided intravenous thrombolysis with alteplase in patients with an acute stroke of unknown onset time. The medication history of all patients randomized in the WAKE-UP trial was documented. The primary safety outcome was any sign of hemorrhagic transformation on follow-up MRI. The primary efficacy outcome was favorable functional outcome defined by a score of 0–1 on the modified Rankin scale at 90 days after stroke, adjusted for age and baseline stroke severity. Logistic regression models were fitted to study the association of prior antiplatelet treatment with outcome and treatment effect of intravenous alteplase.

**Results:**

Of 503 randomized patients, 164 (32.6%) were on antiplatelet treatment. Patients on antiplatelet treatment were older (70.3 vs. 62.8 years, *p* <  0.001), and more frequently had a history of hypertension, atrial fibrillation, diabetes, hypercholesterolemia, and previous stroke or transient ischaemic attack. Rates of symptomatic intracranial hemorrhage and hemorrhagic transformation on follow-up imaging did not differ between patients with and without antiplatelet treatment. Patients on prior antiplatelet treatment were less likely to achieve a favorable outcome (37.3% vs. 52.6%, *p* = 0.014), but there was no interaction of prior antiplatelet treatment with intravenous alteplase concerning favorable outcome (*p* = 0.355). Intravenous alteplase was associated with higher rates of favorable outcome in patients on prior antiplatelet treatment with an adjusted odds ratio of 2.106 (95% CI 1.047–4.236).

**Conclusions:**

Treatment benefit of intravenous alteplase and rates of post-treatment hemorrhagic transformation were not modified by prior antiplatelet intake among MRI-selected patients with unknown onset stroke. Worse functional outcome in patients on antiplatelets may result from a higher load of cardiovascular co-morbidities in these patients.

## Background

Approximately one quarter to one third of all patients receiving intravenous thrombolytic therapy with alteplase (recombinant human tissue plasminogen activator, rt-PA) for the treatment of acute ischemic stroke are on prescribed antiplatelet treatment (APT) [7, 10, 15]. The fact that both antiplatelets and alteplase interfere substantially with the natural hemostasis raises concern about an increased risk of hemorrhagic complications, and in the ARTIS trial a high rate of symptomatic intracerebral hemorrhage (sICH) was observed with simultaneous administration of intravenous alteplase and aspirin [20]. However, neither in current guidelines, nor in clinical practice, is prior APT considered as an exclusion criterion to treatment with alteplase [9, 19].

Several studies have explored the potential association of antiplatelet treatment with hemorrhagic complications in the context of acute thrombolysis for stroke. In this context, it’s important to distinguish between different categories of intracranial hemorrhage following ischemic stroke. Whereas sICH and parenchymal hemorrhage type 2 (PH2) seem to be related to biologic effects of alteplase and clinically relevant for the patients, the haemorrhagic transformation of infarcted brain tissue might just be an epiphenomenon of ischemic damage and reperfusion [14, 16]. Recent meta-analyses unanimously found APT to be significantly associated with higher risk of subsequent intracerebral hemorrhage (ICH) or sICH in patients treated with rt-PA. In contrast, evidence regarding a possible interaction as to functional outcome remains contradictory [4, 7, 15, 17].

WAKE-UP was a randomized, double-blind, placebo-controlled trial on efficacy and safety of MRI-based thrombolysis in wake-up stroke [12]. The trial protocol comprised follow-up MRI at 22–36 h, enabling the detection of even small and asymptomatic ICH following thrombolysis. In the present secondary post hoc analysis of the WAKE-UP trial data, we aimed at studying the efficacy and safety of intravenous thrombolysis among patients on APT.

## Methods

### Study design

Inclusion criteria for the WAKE-UP trial comprised the mismatch between an acute ischemic lesion visible on diffusion weighted imaging (DWI) and no corresponding marked parenchymal hyperintensity on fluid-attenuated inversion recovery (FLAIR). Preceding studies endorsed this mismatch as a surrogate marker of lesion age, indicating that the stroke onset most likely lies within 4.5 h [13]. All patients or their legal representatives provided written informed consent according to national and local regulations and the competent authorities for each study site and the corresponding ethics committee approved the trial. Analysis of MR images and assessment of hemorrhagic transformation was done centrally by a central image reading board. The detailed trial protocol was published together with its main results [12]. For the present post hoc analysis, all data on the medical history of patients randomized in WAKE-UP was reviewed to identify participants with medication history that included current APT prescription. The agents screened for were as follows: Aspirin, Clopidogrel, Dipyridamol, Triflusal (Single-non-aspirin), Ticagrelor, Prasugrel, Ticlopidine and Eptfibatide. Both single and dual therapy were separately recorded. Furthermore, demographic characteristics and clinical data at baseline and follow-up were collected for statistical analysis.

### Outcome measures and endpoints

Primary safety outcome was the occurrence of any hemorrhagic transformation on follow-up imaging 22–36 h after treatment, corresponding to HI-1, HI-2, PH-1 and PH-2 in the Heidelberg bleeding classification [16]. The definition of sICH was according to the Safe Implementation of Thrombolysis in Stroke Monitoring Study (SITS–MOST) local or remote PH-2 plus neurologic deterioration, as indicated by a score on the NIHSS that was higher by 4 points or more than the baseline value or the lowest value between baseline and 24 h, or hemorrhage leading to death. The evaluation of the efficacy results was based on the clinical endpoints as defined in the WAKE-UP study, whereby clinical outcome was assessed at 90 days after stroke. The primary endpoint was favorable outcome defined as a score of 0–1 on the modified Rankin scale (mRS). Secondary endpoint was a shift towards a better functional outcome across the entire range of the mRS (“shift analysis”).

### Statistical analysis

At first, baseline characteristics between patients with and without APT prescription were compared. The statistical analyses of treatment effects were performed in the intention-to-treat population for all patients with available information for clinical endpoints.

For the safety and primary efficacy variables, we calculated unconditional logistic regression models to estimate the odds ratio and its 95% confidence interval (CI). The categorical shift in the distribution of mRS scores was analyzed by fitting a proportional-odds logistic regression model. To investigate the interaction between APT and treatment effect of alteplase, an according interaction term was included in the models. All models fitted were adjusted for the stratification parameters age and NIHSS at randomization. As all analyses were considered exploratory, all tests were carried out with a two-sided alpha level of 5% without correction for multiple comparisons.

## Results

### Patient characteristics

Of 503 randomized patients, 164 patients (32.6%) were on APT. Of these, 134 patients (81.7%) were pretreated with aspirin, 34 patients (20.7%) with clopidogrel, 6 patients (3.7%) with dipyridamole, 1 patient (0.6%) with triflusal, and 11 (6.7%) patients were pretreated with two antiplatelets. For the latter, aspirin was prescribed in all cases, combined with clopidogrel in 7 patients and with dipyridamol in 4 patients. Patients on APT were older (mean age 70.3 vs. 62.8 years, *p* <  0.001), and were more likely to have a medical history of smoking, hypertension, atrial fibrillation, diabetes mellitus type II, hypercholesterolemia, previous ischemic stroke and transient ischemic attacks (all *p* <  0.001, see Table 1). Consistent with this, patients with prior APT showed higher rates of simultaneous pretreatment with antidiabetic drugs, antihypertensives, and statins. Other baseline characteristics were comparable between patients with and without prior APT.

**Table 1:** Baseline characteristics of patients with ischemic stroke and unknown symptom onset

Variable	Prior APT (***n*** = 164)	No prior APT (***n*** = 339)	***P*** Value
Age, mean (SD), years	70.3 (8.2)	62.8 (12.1)	< 0.001
Female sex, No. (%)	60 (36.6)	118 (34.8)	0.692
Time between last seen well and symptom recognition, median (IQR), hours	7.52 (5.00–8.50)	7.00 (4.67–9.50)	0.085
Medical history/risk factors, No. (%)			
Arterial hypertension	120 (73.2)	146 (43.1)	< 0.001
Diabetes mellitus	44 (26.8)	38 (11.2)	< 0.001
Hypercholesterolemia	98 (59.8)	80 (23.6)	< 0.001
Atrial fibrillation	27 (16.5)	32 (9.4)	0.010
History of ischemic stroke	52 (31.7)	16 (4.7)	< 0.001
History of TIA	17 (10.4)	6 (1.8)	< 0.001
Current or former smoker	94 (58)	162 (50)	0.009
NIHSS score, median (IQR)	6 (4–12)	5 (4–9)	0.104
DWI lesion volume at baseline, median (IQR), ml	2.28 (0.80–9.61)	2.23 (0.70–7.70)	0.506

*SD* standard deviation, *IQR* interquartile range, *TIA* transient ischemic attack, *NIHSS* National Institute of Health Stroke Scale, *DWI* diffusion weighted imaging

Information on the primary and secondary efficacy endpoints was available for 490 participants. Of these, 246 and 244 were randomized to treatment with alteplase and placebo, respectively. The proportion of patients on prior APT in the alteplase group (*n* = 75, 30.5%) was comparable to the placebo group (*n* = 86, 35.2%, *p* = 0.290). Information on the primary safety endpoint and the secondary safety endpoint PH-2 hemorrhage was available for 496 participants. Information on the secondary safety endpoint sICH according to SITS–MOST was available for 503 participants.

### Safety outcomes in patients with antiplatelet pretreatment

Prior APT was not associated with higher risk of any radiological hemorrhagic transformation (24.5% vs 23.7%, adjusted OR 0.84, 95% CI 0.63–1.36; *p* = 0.478). There was no significant difference between either groups regarding the occurrence of sICH (2.4% vs 0.6%; adjusted OR 2.84, 95% CI 0.50–16.05; *p* = 0.238). In the group with APT, 5 patients (3.1%) showed PH-2 hemorrhage on follow-up imaging, compared to 6 patients (1.8%) in the group of patients without prior APT (adjusted OR 1.17, 95% CI 0.34–4.09; *p* = 0.806).

### Interaction of antiplatelet pretreatment and treatment safety of alteplase

**Fig. 1:**
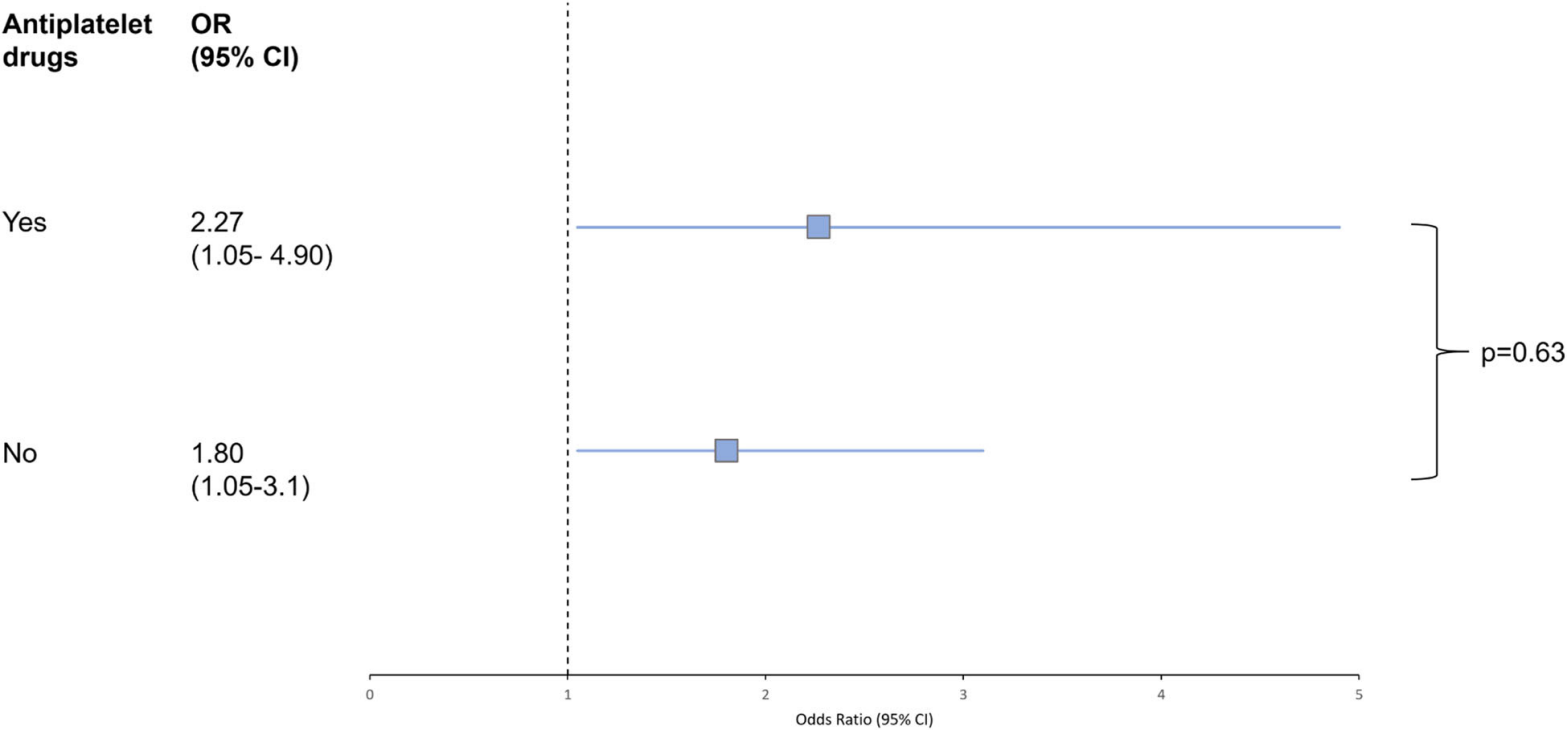
Effect of alteplase on hemorrhagic transformation. Forest plots demonstrate a significant higher chance of developing any sign of hemorrhagic transformation during follow-up for patients treated with alteplase in both patients with prior APT and without. There was no significant interaction in the corresponding unconditional logistic regression model

The general low frequency of sICH and PH-2 hemorrhage in our cohort did not allow for statistical modelling. Treatment with alteplase was associated with higher odds of any hemorrhagic transformation among all randomized patients, but there was no significant interaction effect of thrombolysis with prior APT (*p* = 0.631). The adjusted OR for any hemorrhagic transformation with alteplase was 2.27 (95% CI 1.05–4.90) in patients with prior APT and 1.803 (95% CI 1.05–3.10) in patients without prior APT (Fig. 1).

### Interaction of antiplatelet pretreatment and treatment effect of alteplase

**Fig. 2:**
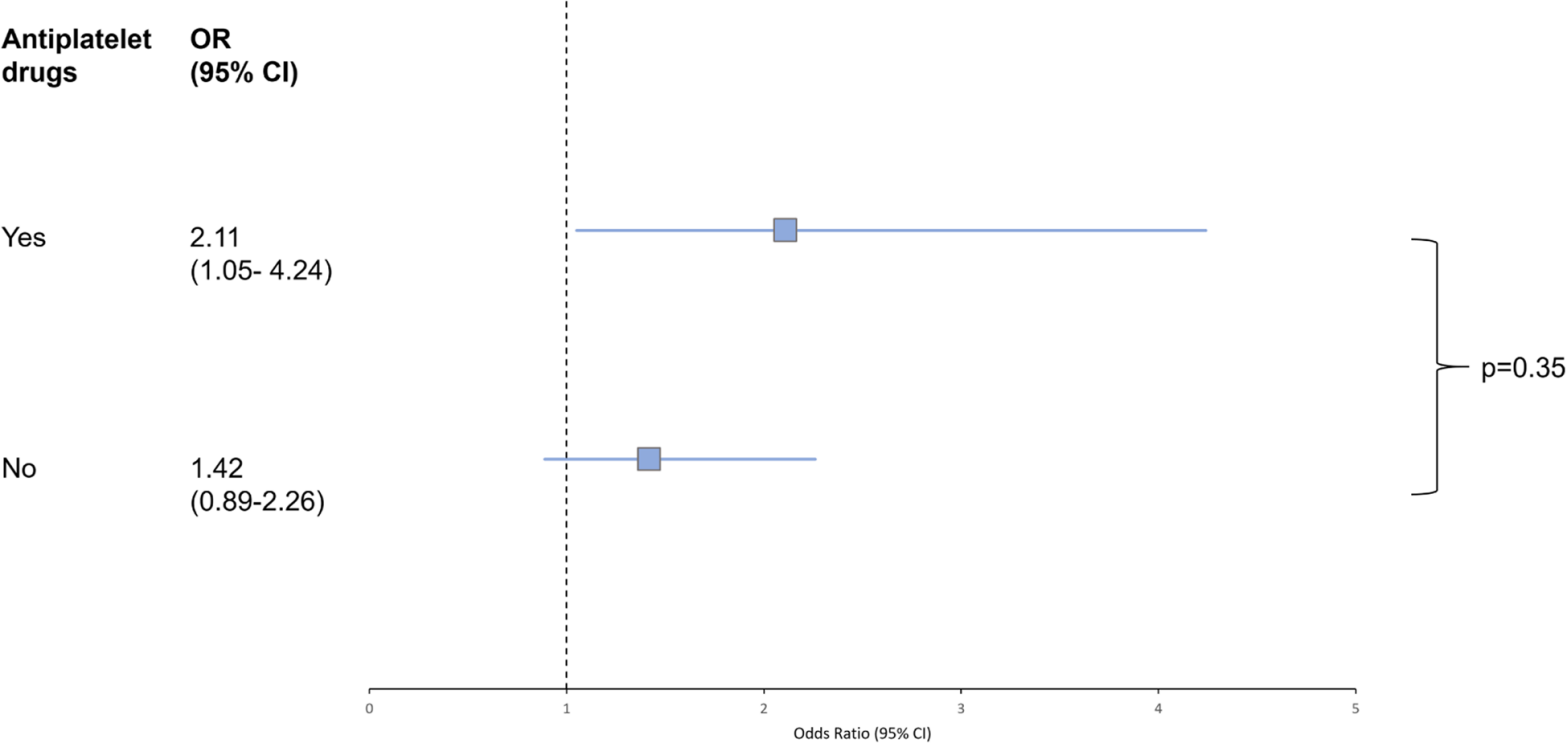
Effect of alteplase on favorable outcome. Forest plots demonstrate a higher chance of a favorable functional outcome for patients treated with alteplase in both patients with prior APT and without. There was no significant interaction in the corresponding unconditional logistic regression model

Patients on APT treatment - independent of thrombolysis treatment group - had lower rates of favorable outcome (37.3 vs. 52.6%; adjusted OR 0.58, 95% CI 0.38–0.89; *p* = 0.014). Treatment with alteplase was associated with higher odds of favorable outcome among all randomized patients with no significant interaction between thrombolysis and prior APT (*p* = 0.355). The adjusted OR for favorable outcome with alteplase was 2.11 (95% CI 1.05–4.24) in patients with prior APT and 1.417 (95% CI 0.89–2.26) in patients without prior APT (Fig. 2).

**Fig. 3:**
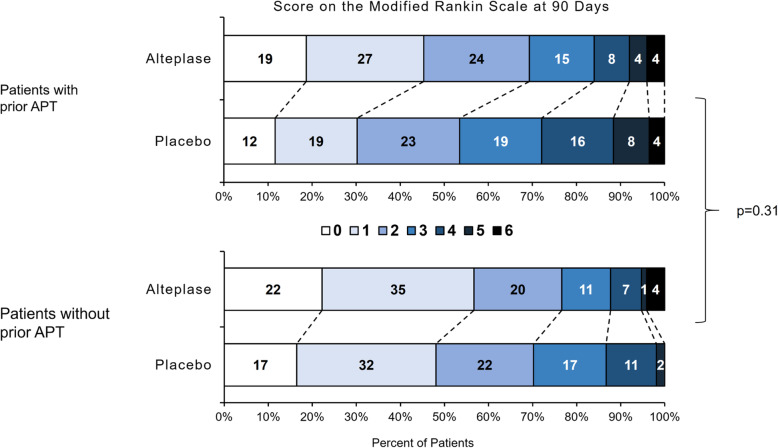
Distribution of modified Rankin scale scores at 90 days after stroke. Distributions of scores on the modified Rankin scale show a favoring of the alteplase group over the placebo group. Modified Rankin scale scores range from 0 to 6 (0, no symptoms; 1, no clinically significant disability; 2, slight disability; 3, moderate disability; 4, moderately severe disability; 5, severe disability; 6, death). Numbers indicate rounded proportions. There was no significant interaction in the corresponding unconditional logistic regression model

Prior APT – independent of treatment group - was associated with a significant shift on the mRS towards worse outcomes (adjusted OR 1.56, 95% CI 1.10–2.23; *p* = 0.014). Treatment with alteplase was associated with a shift on the mRS towards better functional outcome among all randomized patients, but there was no significant interaction of thrombolysis with prior APT (*p* = 0.309). The adjusted OR for a shift on the mRS towards better outcomes with alteplase was 2.00 (95% CI 1.16–3.46) in patients with prior APT and 1.410 (95% CI 0.95–2.09) in patients without prior APT (Fig. 3).

## Discussion

In a post-hoc analysis of the WAKE-UP trial we studied the effect of prescribed antiplatelet therapy prior to stroke on functional outcome and safety outcomes overall, and on the efficacy and safety of intravenous alteplase in acute stroke patients with unknown time of symptom onset. There were two main findings. First, prior antiplatelet therapy was associated with a worse functional outcome independent of treatment, but not with higher rates of symptomatic intracerebral hemorrhage or radiological signs of hemorrhagic transformation. Second, pretreatment with antiplatelets did not alter the beneficial effect or the safety of intravenous alteplase.

In our sample about one in three patients was pretreated with antiplatelets, which is comparable to prior studies on this topic in patients with known time of symptom onset [7, 15]. This reflects the high load of cardiovascular risk factors and co-morbidities in patients with acute stroke, as shown by a history of arterial hypertension in 53%, hypercholesterolemia in 35%, diabetes in 16%, and prior ischemic stroke in 14% of the patients in our study population. Thus, antiplatelets are commonly prescribed among patients treated with intravenous thrombolysis for acute ischemic stroke, and data on the hemorrhagic risk of this thrombolytic treatment in these patients are helpful to inform clinical practice.

Patients on APT treatment had a worse functional outcome in our sample – even after adjustment for age and baseline NIHSS. This might be explained by the higher load of co-morbidities in patients on APT and the known association of more severe co-morbidities and worse functional outcome after stroke [3, 11]. Nevertheless, results from previous studies are contradictory with regard to the association of pretreatment with antiplatelets and outcome of intravenous thrombolysis. Some previous studies in patients with systemic thrombolysis showed a similar association of APT with worse functional outcome as observed in our analysis [4, 7, 10]. In contrast, a meta-analysis of randomized trials of intravenous thrombolysis revealed no significant association of APT and functional outcome after correction for age and stroke severity [15]. These contradictory findings may result from differences in clinical characteristics of the study samples and/or adjusting for varying baseline predictors.

For clinical practice, the question whether APT interacts with the treatment effect of intravenous thrombolysis, is even more important. We did not observe any interaction of prior APT with the treatment effect of alteplase, neither regarding favorable outcome, nor when considering functional outcome across the entire range of the mRS. Intravenous alteplase had a significant beneficial effect on functional outcome within the subgroup of patients with prior APT, a finding which strongly supports the current practice that does not consider antiplatelet treatment as a contraindication for intravenous thrombolysis. To the best of our knowledge, this explicit exclusion of a statistical interaction with the effects of thrombolysis isnovel and adds substantially to the available research on pre-treatment with antiplatelets and alteplase.

The main concern regarding pretreatment with antiplatelets in patients receiving intravenous alteplase refers to ICH, as there is in general an increased risk of ICH in stroke patients on prior APT, independently of thrombolysis: in a Korean registry of 10,433 patients with acute and subacute ischemic stroke, there were higher adjusted odds of hemorrhagic transformation on MRI at presentation in patients on prior APT [8].

The numbers of radiologically severe and symptomatic ICH after intravenous thrombolysis in our study were small, most likely an artefact of the general low severity in the MRI-selected population of the WAKE-UP trial. Therefore, we decided to study any hemorrhagic transformation detected by follow-up MRI after 22–36 h. Among all randomized patients, intravenous thrombolysis was significantly associated with a higher risk of developing any hemorrhagic transformation, which is a well-known phenomenon linked to the biological effects of alteplase. However, in our study patients on APT treatment did not show higher rates of hemorrhagic transformation, nor did prior APT interact with alteplase with regard to the rates of hemorrhagic transformation. These findings are in line with a metanalysis, in which no association of APT with sICH was observed after correction for patient-level characteristics [15]. In contrast, other previous studies found prior APT to be associated with increased rates of ICH [10, 17] and sICH [3, 4, 6, 7, 10, 18] in patients treated with thrombolysis. Again, these contradictory findings may result from differences in clinical characteristics of the study samples.

There are limitations to our study. The number of clinical relevant symptomatic intracranial hemorrhages was small in both groups, so our study was underpowered to detect differences between the groups with regard to these important safety outcomes. Therefore, we can’t exclude a potential difference in both groups, nor can we exclude an interaction with alteplase with respect to safety.

Moreover, due to the small numbers of patients taking more than one antiplatelet drug, we were not able to study the potential effect of double antiplatelet treatment on safety and efficacy of treatment with intravenous alteplase in our cohort. Whereas a post-hoc analysis of the Safe Implementation of Treatments in Stroke (SITS) International Stroke Thrombolysis Register found a combination of aspirin and clopidogrel associated with higher rates of sICH, a recent meta-analysis, however, revealed no significant risk of sICH, 3-month mortality or worse favourable outcome after 3 months in these patients [1, 5, 6]. The numbers of patients taking other antiplatelet drugs than aspirin was also small in our cohort, so that we could not study specific risks of different antiplatelet agents. However, a previous study with a comparable distribution of aspirin, clopidogrel and other antiplatelet agents found no specific differences on severity of stroke at presentation, in-hospital mortality and mRS at discharge [2].

## Conclusion

To summarize, in the randomized controlled WAKE-UP trial, treatment with intravenous alteplase was safe and efficient in patients with unknown onset stroke on prior antiplatelet therapy, even though the latter might be a predictor of worse functional outcome. Intravenous thrombolysis should not be withheld from patients with unknown onset acute ischemic stroke on prior antiplatelet therapy.

## Data Availability

Due to the nature of this research, participants of this study did not agree for their data to be shared publicly, so supporting data is not available.

## Abbreviations

APT: AntiPlatelet Treatment
ICH: IntraCerebral Hemorrhage
MRI: Magnetic Resonance Imaging
mRS: modified Rankin Scale
NIHSS: National Institutes of Health Stroke Scale
OR: Odds Ratio
sICH: symptomatic IntraCerebral Hemorrhage
